# Passage of the Bill to Regulate the Rank of the Staff of the U. S. Army

**Published:** 1874-06

**Authors:** 


We extract the following from the Washington *Evening Star* of June 25:

The bill to reorganize the staff of the army was among those passed during the closing minutes of the session. Among the provisions of this bill is one raising the chief medical purveyor to the rank of colonel. Heretofore this officer has had the rank of lieutenant colonel. Immediately after signing the bill the President sent to the Senate the name of Lieutenant Colonel J. H. Baxter, now chief medical purveyor of the army. Colonel S. V. Benet, of the ordnance corps, was at the same time sent for promotion to the rank of brigadier general and chief of ordnance. Both these nominations were confirmed just previous to he final adjournment. The p ssage of the bill is considered a great victory by the friends of the army.

